# TLR4 Signaling and Heme Oxygenase-1/Carbon Monoxide Pathway Crosstalk Induces Resiliency of Myeloma Plasma Cells to Bortezomib Treatment

**DOI:** 10.3390/antiox11040767

**Published:** 2022-04-12

**Authors:** Grazia Scandura, Cesarina Giallongo, Fabrizio Puglisi, Alessandra Romano, Nunziatina Laura Parrinello, Tatiana Zuppelli, Lucia Longhitano, Sebastiano Giallongo, Michelino Di Rosa, Giuseppe Musumeci, Roberto Motterlini, Roberta Foresti, Giuseppe Alberto Palumbo, Giovanni Li Volti, Francesco Di Raimondo, Daniele Tibullo

**Affiliations:** 1 Division of Hematology, Department of General Surgery and Medical-Surgical Specialties, A.O.U. “Policlinico-Vittorio Emanuele”, University of Catania, 95123 Catania, Italy; gra.scandura@gmail.com (G.S.); puglisi.fabri@gmail.com (F.P.); sandrina.romano@gmail.com (A.R.); lauraparrinello@tiscali.it (N.L.P.); diraimon@unict.it (F.D.R.); 2 Department of Scienze Mediche Chirurgiche e Tecnologie Avanzate “G.F. Ingrassia”, University of Catania, 95123 Catania, Italy; cesarina.giallongo@unict.it; 3 Department of Biomedical and Biotechnological Sciences, University of Catania, 95123 Catania, Italy; tatiana.zuppelli@gmail.com (T.Z.); lucialonghitano@hotmail.it (L.L.); sebastiano.giall@gmail.com (S.G.); mdirosa@unict.it (M.D.R.); g.musumeci@unict.it (G.M.); d.tibullo@unict.it (D.T.); 4 Faculty of Health, University Paris-Est Créteil, INSERM, IMRB, 94010 Créteil, France; roberto.motterlini@inserm.fr (R.M.); roberta.foresti@inserm.fr (R.F.)

**Keywords:** multiple myeloma, TLR4/HO-1 crosstalk, mitochondria, bortezomib

## Abstract

Relapse in multiple myeloma (MM) decreases therapy efficiency through unclear mechanisms of chemoresistance. Since our group previously demonstrated that heme oxygenase-1 (HO-1) and Toll-like receptor 4 (TLR4) are two signaling pathways protecting MM cells from the proteasome inhibitor bortezomib (BTZ), we here evaluated their cross-regulation by a pharmacological approach. We found that cell toxicity and mitochondrial depolarization by BTZ were increased upon inhibition of HO-1 and TLR4 by using tin protoporphyrin IX (SnPP) and TAK-242, respectively. Furthermore, the combination of TAK-242 and BTZ activated mitophagy and decreased the unfolded protein response (UPR) survival pathway in association with a downregulation in HO-1 expression. Notably, BTZ in combination with SnPP induced effects mirroring the treatment with TAK-242/BTZ, resulting in a blockade of TLR4 upregulation. Interestingly, treatment of cells with either hemin, an HO-1 inducer, or supplementation with carbon monoxide (CO), a by-product of HO-1 enzymatic activity, increased TLR4 expression. In conclusion, we showed that treatment of MM cells with BTZ triggers the TLR4/HO-1/CO axis, serving as a stress-responsive signal that leads to increased cell survival while protecting mitochondria against BTZ and ultimately promoting drug resistance.

## 1. Introduction

Multiple myeloma (MM) is a malignant hematologic disease characterized by the clonal proliferation of malignant plasma cells (PCs). Despite recent advances in MM treatment, most patients eventually relapse, with the duration of response decreasing with each line of therapy [1]. Therefore, additional studies are warranted to elucidate the molecular mechanisms underlying the development of drug resistance in this pathological condition to further develop more effective treatments and quality outcomes in myeloma patients.

Toll-like receptor 4 (TLR4) is one of the most characterized TLRs, and its activation triggers two different downstream pathways, namely, MyD88-dependent and MyD88-independent signaling. The former activates the mitogen-activated protein kinase (MAPK) and nuclear factor-kappa B (NF-kB) pathways, leading to an inflammatory phenotype [2], whereas the second pathway involves Toll/IL-1R domain-containing adapter-inducing interferon-β (TRIF) and Toll receptor-associated molecule (TRAM) mediating resistance to virus infection [3]. Recently, different clinical trials have been performed, aiming to investigate the potential of TLR4 inhibitor TAK-242 to decrease the inflammatory response in severe sepsis and acute alcoholic hepatitis (ClinicalTrials.gov Identifier: NCT00143611 and NCT04620148). TLR4 and MyD88 have been demonstrated to be overexpressed in PCs resistant to bortezomib, a proteasome inhibitor [4]. In particular, activation of TLR4 by lipopolysaccharide (LPS) influences mitochondrial dynamics in myeloma PCs and contributes to bioenergetic changes in PCs resistant to proteasome inhibitors including an increase in mitochondrial mass and an increased reliance on mitochondrial respiration [5,6,7,8]. Consistently, inhibition of TLR4 restores pharmacological responses of resistant cells to bortezomib, impairing their mitochondrial dynamics and metabolism associated with a decrease of the hexosamine biosynthetic pathway [4]. One of the hallmarks of MM cells is the excessive production of monoclonal immunoglobulin associated with a constitutive expression of endoplasmic reticulum (ER) stress survival factors ensuring their secretory cell function [9]. To cope with ER stress, cells activate the unfolded protein response (UPR), which increases the biosynthetic capacity and decreases the biosynthetic burden of the ER to maintain cellular homeostasis. To this regard, TLR4 activates the IRE1α (inositol-requiring enzyme 1 alpha) arm of the UPR system and its downstream target XBP1 (splice X-box binding protein 1), a pro-survival transcription factor regulating genes responsible for protein folding and degradation during the UPR, being crucial for the development of antibody-secreting PCs [10,11]. Activation of TLR4 signaling favors myeloma PC proliferation and decreases the pro-apoptotic efficacy of bortezomib through the suppression of ER-stress-induced apoptosis [12] but at the same time leads to activation of the nuclear factor erythroid 2–related factor 2 (Nrf2), which in turn controls the expression of several detoxifying and antioxidant enzymes including heme oxygenase-1 (HO-1) [13]. HO-1 is an endoplasmic-reticulum-anchored enzyme involved in heme catabolism, exerting potent antioxidant properties exploited by cancer cells to resist to oxidative stress [14]. HO-1 increased expression is associated with both an anti-inflammatory response and increased mitochondrial biogenesis [15]. Several stress conditions of a pharmacological agent may induce HO-1 expression, and interestingly, our group showed that bortezomib treatment significantly upregulated HO-1 in myeloma PCs [16]. The protective role of HO-1 relies on its ability to decrease the expression of key pathways involved in ER stress [17] such as PERK (protein-kinase-like endoplasmic reticulum kinase), p-eIF2-α (eukaryotic initiation factor 2), ATF4 (activating transcription factor 4), and CHOP (C/EBP homologous protein) [18]. In addition, HO-1 upregulation is associated with ER stress in MM cells exposed to bortezomib [16]. In particular, PERK directly phosphorylates Nrf2, promoting dissociation from its repressor Keap-1 with the subsequent activation of the Nrf2/HO-1 axis [19], while Nrf2-ATF4 dimers bind to HO-1 promoter, thus regulating its expression [20]. On the other hand, Nrf2/HO-1 negatively regulates CHOP expression, which is positively regulated by ATF4 [18]. Interestingly, increase of HO-1 expression in MM cells treated with bortezomib is accompanied by upregulation of TLR4 [4], indicating a potential connection between these two signaling pathways that may synergistically work to resolve ER stress and maintain mitochondrial integrity.

The aim of the present study was to evaluate the possible crosstalk between the TLR4 signaling and HO-1/CO pathway in MM cells and its impact on bortezomib chemoresistance.

## 2. Materials and Methods

### 2.1. Cell Culture and Treatments

Human myeloma cell lines (HMCLs) were grown in RPMI 1640 medium supplemented with 10% (for NCI-H929 and OPM2) and 20% (for U266) fetal bovine serum and 1% penicillin–streptomycin. NCI-H929 and U266 cell lines were obtained from ATCC (Mannas, VA, USA). OPM2 cell line was purchased from Leibniz Institute DSMZ German Collection of Microorganisms and Cell Cultures GmbH (Braunschweig, Germany).

To activate TLR4, cells were treated with LPS (Sigma-Aldrich, Mylan, Italy). The TLR4 inhibitor TAK-242 and the HO-1 inhibitor tin protoporphyrin IX (SnPP) were purchased from Sigma-Aldrich and Cayman (Ann Arbor, MI, USA), respectively. Hemin was obtained from Sigma-Aldrich and dissolved in 0.1 M NaOH. Commercially available bortezomib (BTZ) was used. CORM3 and CORM-A1 were purchased from Sigma-Aldrich (St. Louis, MI, USA).

### 2.2. Flow Cytometry

Reactive oxygen species (ROS) were detected using 2′,7′-dichlorodihydrofluorescein acetate (H2-DCF; Sigma-Aldrich, St. Louis, MO, USA), and fluorescence intensity was measured according to the fluorescence detection conditions of FITC by using a MACSQuant Analyzer (Miltenyi Biotech, North Rhine-Westphalia, Germany), as already reported [21].

Moreover, to measure changes in the mitochondrial mass, cells were reacted with 200 nM MitoTracker Red CMXRos probe (Thermo Fisher Scientific, Milan, Italy) for 30 min at 37 °C, according to the manufacturer′s instructions. After being washed twice, cells were treated. Then, 24 h later, they were analyzed by flow cytometry.

### 2.3. Immunofluorescence

Immunofluorescent analysis was performed as already described [21]. After treatment, cells were adhered to slides by cytospin and subsequently fixed with 4% formaldehyde for 20 min at room temperature. The slides were then incubated overnight at 4 °C with the primary antibody against Nrf2 (anti-rabbit; Santa Cruz Biotechnology, Dallas, TX, USA) and NF-kB (anti-rabbit; Santa Cruz Biotechnology) at a dilution of 1:100. The slides were mounted with medium containing DAPI (4,6-diamidino-2-phenylindole) to visualize nuclei.

To investigate mitophagy, cells were labeled with 200 nM MitoTracker Red CMXRos probe (M7512, Thermo Fisher Scientific, Rodano, Milan, Italy) before the treatment. Cells were subsequently incubated with primary antibody against LC3-II-rabbit (L7543, Sigma-Aldrich, Milan, Italy) at 1:100 dilution. The fluorescent images were obtained using a Zeiss Axio Imager Z1 Microscope with Apotome 2 system (Zeiss, Milan, Italy).

### 2.4. Western Blot Analysis

Briefly, Western blot analysis was performed as previously reported [21]. Protein was detected using primary antibodies against human TLR4 (sc-293072, Santa Cruz Biotechnology, Santa Cruz, CA, USA), p38, phospho-p38, ERK, phospho-ERK (#9212, #9211, #4377, #9102 Cell Signaling Technology, Danvers, MA, USA), PINK1, GAPDH and β-actin (ab8226, ab23707, ab181602, Abcam, Milan, Italy), PERK, IRE1 α, GRP78, or HO1 (BML-HC3001-0025, Enzo Life Sciences, Milan, Italy) overnight at 4 °C. The blots were visualized using an Odyssey Infrared Imaging Scanner (Licor, Milan, Italy), and protein levels were quantified by densitometric analysis of antibody responses. Data were normalized to protein levels of β-actin or GAPDH.

### 2.5. RT-qPCR

The relative transcription of specific genes was determined by RT-qPCR using Brilliant III Ultra-Fast SYBR Green QPCR Master Mix (Agilent Technologies, Milan, Italy) and a 7900HT Fast Real-Time PCR System (Thermo Fisher, Waltham, MA, USA), as previously reported. Expression of the following human genes was evaluated: *CHOP* (Gene ID: 1649) (Fw: ACCTCCTGGAAATGAAGAGGAAG; Rw: CAGTCAGCCAAGCCAGAGAA), *B2M* (Gene ID: 567) (Fw: AGCAGCATCA TGGAGGTTTG; Rw: AGCCCTCCTA GAGCTACCTG); *GAPDH* (Gene ID: 2597) (Fw: AATGGGCAGC CGTTAGGAAA; Rw: GCCCAATAC GACCAAATCAGAG). For each sample, the relative expression level of the mRNA of interest was determined by comparison with the control housekeeping genes B2M and GAPDH using the 2^−∆∆Ct^ method.

### 2.6. Statistical Analysis

Statistical analyses were performed with GraphPad Prism 5.01 (GraphPad Software Inc., San Diego, CA, USA. https://www.graphpad.com, accessed on 28 January 2022). Differences between groups were determined by Student’s *t*-test (to compare 2 groups) or ANOVA (with Fisher’s protected least squares as the post hoc test to compare more than 2 groups), and *p*-values < 0.05 were considered statistically significant.

## 3. Results

### 3.1. TLR4 Inhibition Improved the Efficacy of BTZ through Increased Oxidative Stress Coupled with Mitophagy

**Figure 1:**
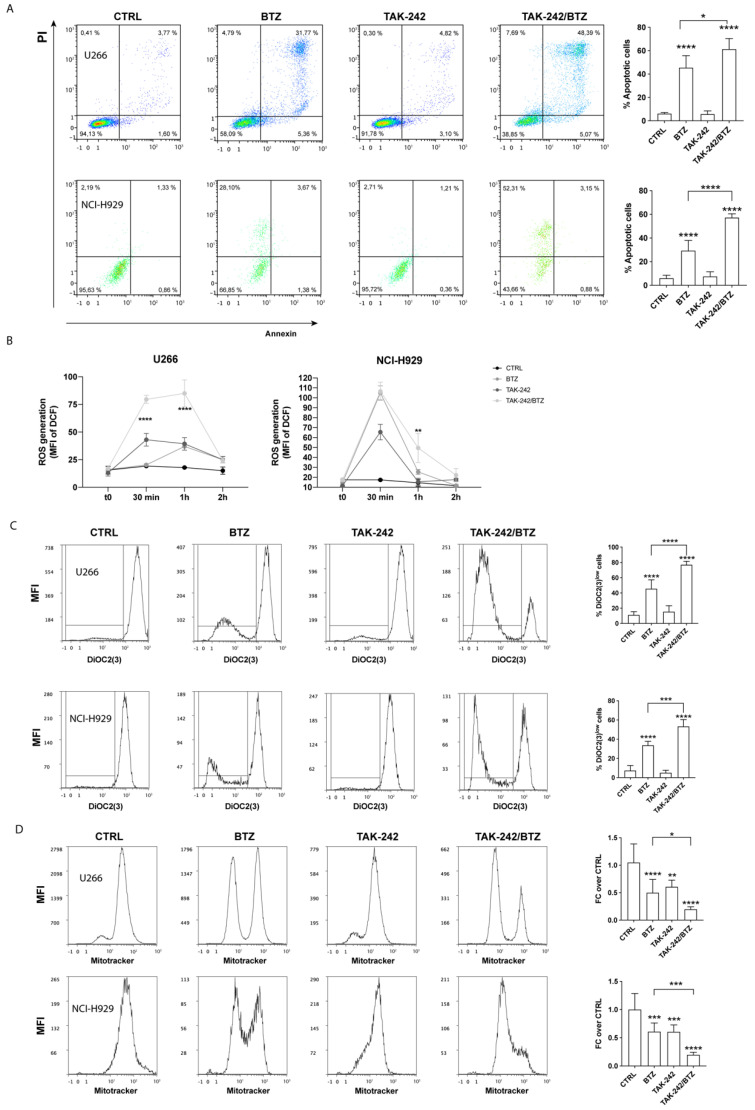
TLR4 inhibition increased BTZ cytotoxicity by promoting oxidative stress coupled with mitophagy. (**A**) Representative dot plots of the effect of TAK-242, BTZ, and TAK-242/BTZ treatment on the viability of U266 and NCI-H929 cells are shown. The graphs (right panels) show the mean values of the percentage of apoptotic cells after annexin V–FITC and PI staining. (**B**) Reactive oxygen species production during drug treatment was measured in MM cell lines by the oxidation of 2′,7′-dichlorofluorescein (DCF-DA) using flow cytometry. (**C**) Mitochondrial membrane potential was assessed by using DiOC2(3) staining. Representative histograms of a flow cytometry analysis are shown (left panels). (**D**) Flow cytometric analysis of mitochondrial mass 24 h post-treatment was determined by using MitoTracker Red fluorescence. Representative histograms (left panels) are shown. Data are expressed as mean MFI ± SEM of *n* ≥ 4 biological replicates; * *p* < 0.05; ** *p* < 0.01; *** *p* < 0.001, **** *p* < 0.0001.

**Figure 2:**
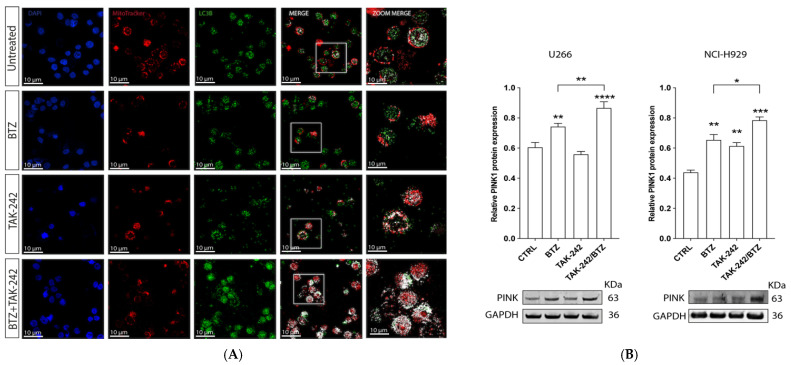
TLR4 inhibition increased BTZ-induced mitophagy activation. (**A**) Immunofluorescence for the colocalization of LC3 (green) and mitochondria (stained by using MitoTracker red) after drug treatments in myeloma cells. (**B**) Analysis of PINK1 expression after drug treatments for 24 h. GAPDH protein was used as total protein loading reference. For analysis, the optical density of the bands was measured using Scion Image software. Data are expressed as mean MFI ± SEM of *n* ≥ 4 biological replicates; * *p* < 0.05; ** *p* < 0.01; *** *p* < 0.001, **** *p* < 0.0001.

To evaluate how TLR4 signaling contributes to the acquisition of a BTZ resistant phenotype, we used the inhibitor TAK-242, which potently suppresses ligand-dependent and -independent TLR4 pathways by binding to the intracellular receptor domain. MM cell lines were pre-treated with 10 µM TAK-242 for 1 h before the addition of BTZ (15 nM). The combination of TAK-242 and BTZ significantly increased the cytotoxic effect of BTZ, as indicated by an increased number of apoptotic cells: 15.9 ± 1.2% and 27.9 ± 5.4% in TAK-242/BTZ-treated cells compared to BTZ-treated alone, respectively, in U266 and NCI-H929 cell lines (*p* < 0.05 and *p* < 0.0001, respectively; Figure 1A). Furthermore, we observed that pretreatment with the TLR4 inhibitor significantly increased the amount of BTZ-induced ROS. As shown in Figure 1B, the median fluorescent intensity (MFI) of H2-DCF increased from 20.3 ± 0.9 in BTZ treated cells to 79 ± 3.6 after 30 min (*p* < 0.0001) in U266 and from 25.4 ± 2.5 to 49.5 ± 14.5 after 1 h (*p* < 0.01) in NCI-H929. As high levels of ROS are associated with impaired mitochondrial function, we evaluated the effects of TAK-242/BTZ combination in comparison with BTZ treatment alone. Pre-treatment with TAK-242 increased the percentage of cells containing depolarized mitochondria of about 31.4 ± 7% and 19.5 ± 3.1%, respectively, in U266 and NCI-H929 cells (*p* < 0.0001 and *p* < 0.001, respectively; Figure 1C). This strong mitochondrial depolarization was accompanied by a decrease in mitochondrial mass (Figure 1D). Indeed, after TAK-242/BTZ treatment, MitoTracker-MFI value decreased by about 0.3 ± 0.19- and 0.4 ± 0.1-fold, respectively, in U266 and NCI-H929 cells compared to BTZ-treated ones (*p* < 0.05 and *p* < 0.001, respectively). Consistent with these data, we also observed an increased expression of in PINK1 (PTEN-induced kinase 1) after TAK-242/BTZ treatment compared to BTZ-treated cells (*p* < 0.01 in U266 and *p* < 0.05 in NCI-H929) (Figure 2). Notably, exposure of NCI-H929 to TAK-242 alone significantly increased PINK1 expression compared to control cells (*p* < 0.01), in agreement with the strong reduction of mitochondrial mass observed above in TAK-242-treated cells. As the reduction of mitochondrial mass associated with PINK1 overexpression was higher in MM cells treated with TAK-242/BTZ, we next evaluated if this drug combination activated mitophagy, a key process in the mitochondrial quality control aimed at the removal of dysfunctional mitochondria. We found that pre-treatment with TAK-242 strongly increased BTZ-induced mitophagy, as demonstrated by the colocalization of LC3 protein (a constituent of the autophagosome) with Mitotracker-stained mitochondria (Figure 2) (about 38-fold higher than BTZ-treated cells; *p* < 0.001).

### 3.2. TLR4 Signaling Induced HO-1 Expression through Nrf2 Activation in Myeloma Plasma Cells (or Promoted Nrf2/HO-1 Pathway in Myeloma Plasma Cells)

**Figure 3:**
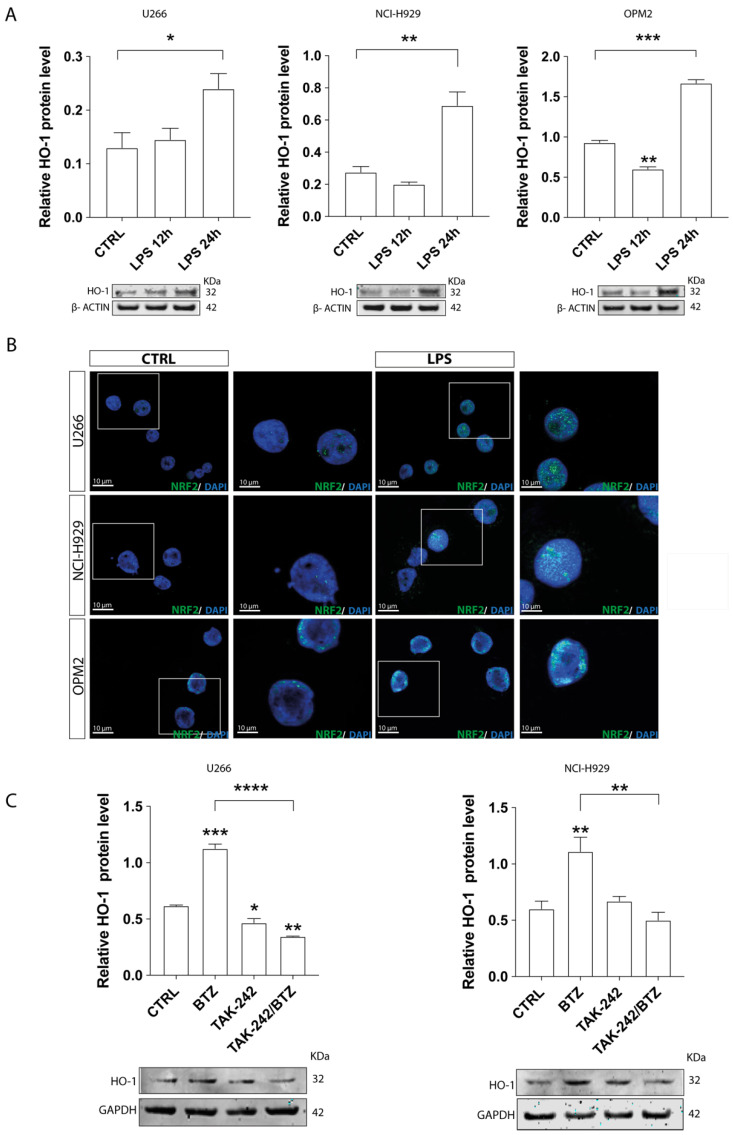
TLR4 modulated HO-1 expression. (**A**) Western blot analysis of HO-1 expression in myeloma cell lines after LPS treatment. β-actin protein was used as total protein loading reference. (**B**) Immunofluorescence of Nrf2 (green) nuclear translocation after LPS treatment in myeloma cell lines. (**C**) Western blot analysis of HO-1 expression in myeloma cell lines 24 h after drug treatments. GAPDH protein was used as total protein loading reference. For Western blot analysis, the optical density of the bands was measured using Scion Image software. Data are expressed as mean MFI ± SEM of *n* ≥ 4 biological replicates; * *p* < 0.05; ** *p* < 0.01; *** *p* < 0.001, **** *p* < 0.0001.

To test the involvement of TLR4 signaling on HO-1 expression, we treated HMCLs with 2 μg/mL LPS for 12 and 24 h and then assessed the levels of HO-1 protein by Western blotting. LPS induced HO-1 expression after 24 h in all the three MM cell lines tested (*p* < 0.05 in U266, *p* < 0.01 in NCI-H929, and *p* < 0.001 in OPM2 compared to control; Figure 3A). Since HO-1 protein expression is regulated through Nrf2 binding to its antioxidant response elements (ARE) [22], we then tested the effect of LPS on Nrf2 nuclear translocation, a necessary step for HO-1 upregulation. As expected, LPS caused Nrf2 accumulation in the nucleus of HMCLs (Figure 3B), suggesting that activation of the NrF2/HO-1 signaling axis occurs when the TLR4 pathway is activated in MM cells.

### 3.3. TAK-242/BTZ Cotreatment Decreased the Expression of HO-1 and UPR Survival Pathways

**Figure 4:**
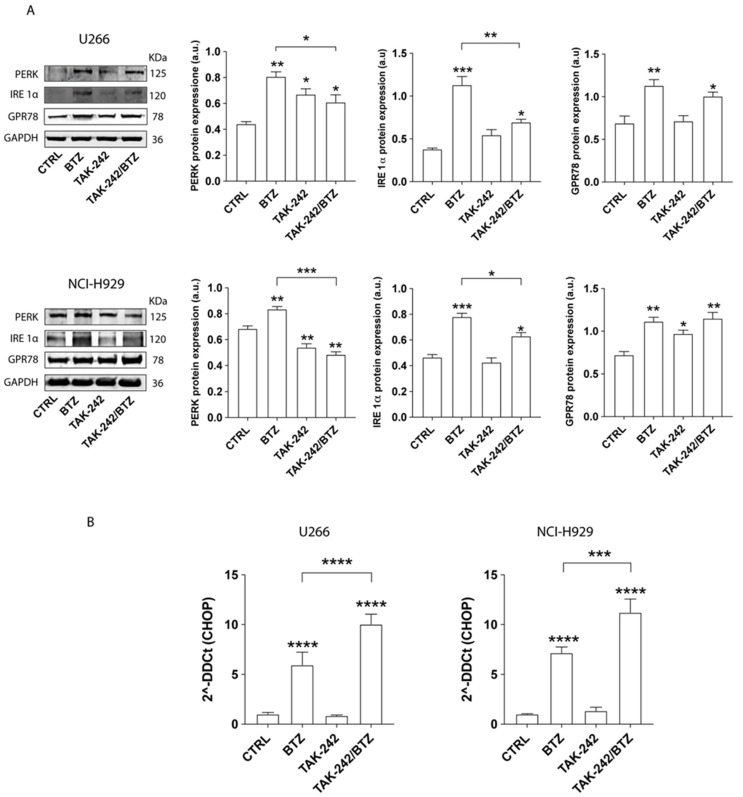
TLR4 inhibition decreased BTZ-induced ER stress. (**A**) Western blot analysis of expression of ER stress protein markers (PERK, IRE1α, GPR78) in myeloma cell lines after drug treatments. GAPDH protein was used as total protein loading reference. For analysis, the optical density of the bands was measured using Scion Image software. (**B**) Relative mRNA expression of CHOP after drug treatments. Calculated value of 2^−∆∆Ct^ in untreated cells was 1. Data are expressed as mean MFI ± SEM of *n* ≥ 4 biological replicates; * *p* < 0.05; ** *p* < 0.01; *** *p* < 0.001, **** *p* < 0.0001.

Given our findings, we then examined the impact of TAK-242/BTZ on HO-1 expression. As previously demonstrated by our group [18], BTZ treatment increased HO-1 expression in comparison with untreated cells (*p* < 0.001 in U266 cells and *p* < 0.01 in NCI-H929; Figure 3C). Notably, the pre-treatment with TAK-242 significantly decreased BTZ-induced HO-1 upregulation (*p* < 0.0001 and *p* < 0.01 compared to BTZ treated cells, respectively, in U266 and NCI-H929). Interestingly, TAK-242/BTZ treatment also decreased the overexpression of ER stress markers PERK (*p* < 0.05 and *p* < 0.001, respectively, in U266 and NCI-H929) and IRE1α (*p* < 0.05 in both cell lines) compared to cells treated with BTZ alone (Figure 4A). GRP78/BIP expression did not change between MM cells treated with BTZ or its combination with TAK-242. To better demonstrate that inhibition of BTZ-induced upregulation of ER transmembrane signaling markers (PERK and IRE1α) is linked to an increased apoptotic effect of BTZ by impairing the UPR survival pathway, we next evaluated the expression of *CHOP*, one of the UPR downstream effectors that promotes apoptosis. In both MM cell lines, TAK-242/BTZ cotreatment induced higher mRNA expression of *CHOP* (*p* < 0.001 compared to BTZ alone; Figure 4B).

### 3.4. Inhibition of HO-1 Enzymatic Activity Improved the Pharmacological Response of Myeloma PCs to BTZ by Increasing ER Stress and Mitochondrial Damage

**Figure 5:**
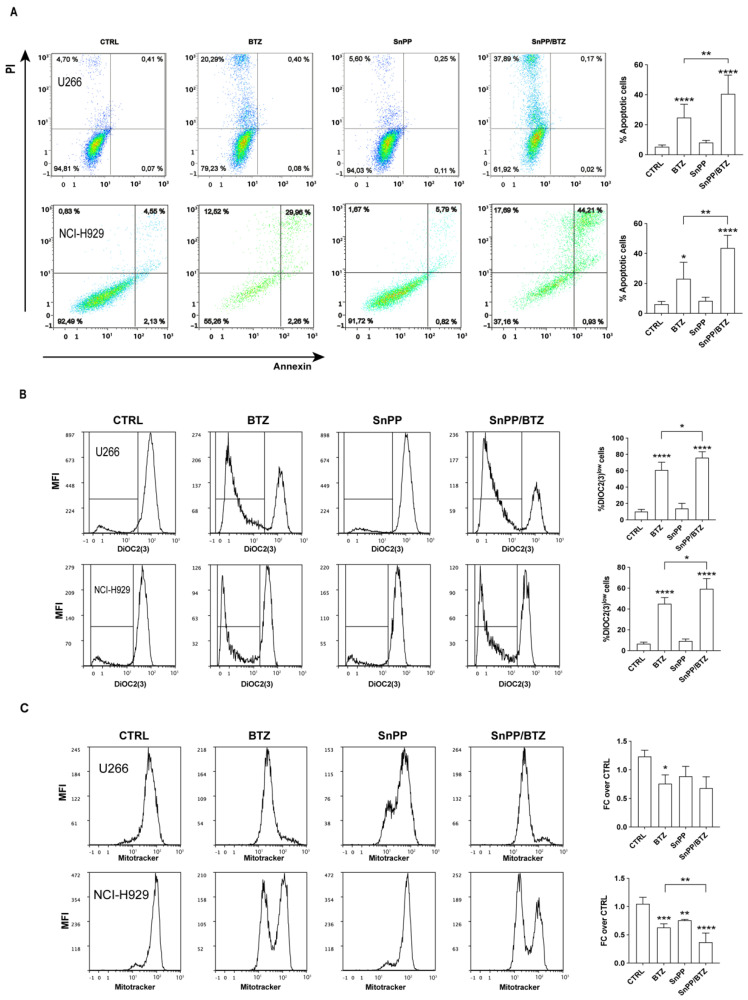
HO-1 enzymatic inhibition increased BTZ cytotoxicity by rising mitochondrial depolarization. (**A**) Representative dot plots of the effect of SnPP, BTZ, and SnPP/BTZ treatment on the viability of U266 and NCI-H929 cells are shown. The graphs (right panels) show the mean values of the percentage of apoptotic cells after annexin V–FITC and PI staining. (**B**) Mitochondrial membrane potential was evaluated after drug treatment. Representative histograms of a flow cytometric analysis are shown (left panels). (**C**) Flow cytometry analysis of mitochondrial mass 24 h post-treatment. Representative histograms (left panels) are shown. Data are expressed as mean MFI ± SEM of *n* ≥ 4 biological replicates; * *p* < 0.05; ** *p* < 0.01; *** *p* < 0.001; **** *p* < 0.0001.

In order to investigate if HO-1 downregulation induced by the combination of TAK-242 with BTZ could contribute to mitochondrial damage observed in MM cells cotreated with these drugs, the inhibitor of HO-1 enzymatic activity SnPP was used in additional experiments. Myeloma PCs were pre-treated with 10 µM SnPP for 24 h prior to addition of BTZ. We found that U266 and NCI-H929 exposed to SnPP/BTZ showed a significant increase in annexin V+/PI+ cells (15.9 ± 3.5% and 20.6 ± 2.6%, respectively; *p* < 0.01) compared to BTZ-treated cells alone (Figure 5A), indicating that blockade of HO-1 leads to increased cell apoptosis caused by BTZ.

To evaluate if the combination of BTZ with SnPP affects mitochondrial function, we further assessed their effects on mitochondrial membrane potential. SnPP/BTZ treatment increased the percentage of depolarized cells to 14.1 ± 3.4% and 13.2 ± 3.4%, respectively, in U266 and NCI-H929 cells (*p* < 0.05 compared to BTZ alone; Figure 5B). SnPP alone did not have any effect on mitochondrial polarization. Concerning the mitochondrial mass content, we found that treatment with SnPP/BTZ decreased MitoTracker-MFI values only in NCI-H929 cells (*p* < 0.01 compared to BTZ alone; Figure 5C) but not in in U266 cells. In both cell lines, SnPP alone significantly decreased MitoTracker-MFI value compared to untreated cells.

**Figure 6:**
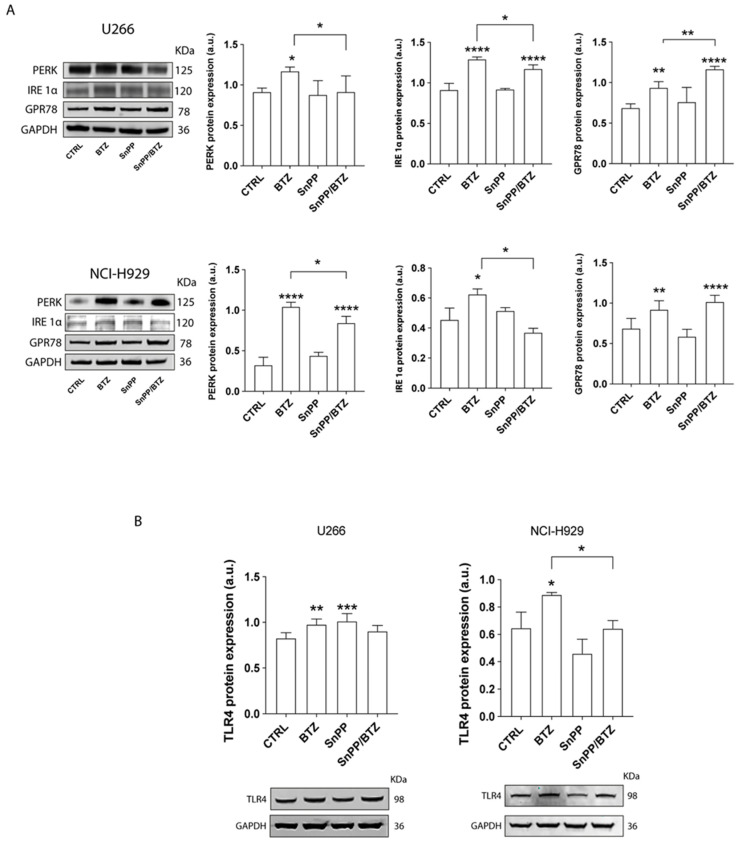
HO-1 enzymatic inhibition decreased BTZ-induced ER stress. (**A**,**B**) Analysis of expression of ER stress protein markers (PERK, IRE1α, GPR78) and TLR4 after SnPP treatment alone or in combination with BTZ. GAPDH protein was used as total protein loading reference. For analysis, the optical density of the bands was measured using Scion Image software. Data are expressed as mean MFI ± SEM of *n* ≥ 4 biological replicates; * *p* < 0.05; ** *p* < 0.01; *** *p* < 0.001; **** *p* < 0.0001.

We next investigated the effect of the inhibition of HO-1 enzymatic activity on UPR response. SnPP pre-treatment significantly decreased the BTZ-induced PERK and IRE1α in both U266 and NCI-H929 (*p* < 0.05; Figure 6A). Expression of GRP78 did not change in NCI-H929 cells exposed to SnPP/BTZ compared to the ones treated with BTZ. In contrast, its expression increased in U266 cell line after SnPP/BTZ treatment compared to BTZ alone (*p* < 0.01).

### 3.5. SnPP Alone Did Not Affect the Mitochondrial Polarization Status

We then asked whether inhibition of HO-1 enzymatic activity could affect TLR4 expression in MM cells. As shown in Figure 6B, in pretreatment of NCI-H929 cells with SnPP inhibited upregulation of TLR4 induced by BTZ (*p* < 0.05 compared to BTZ treated cells), while no differences in TLR4 expression were observed in U266 cells.

### 3.6. HO-1 Regulated TLR4 Expression through Carbon Monoxide (CO) Production

**Figure 7:**
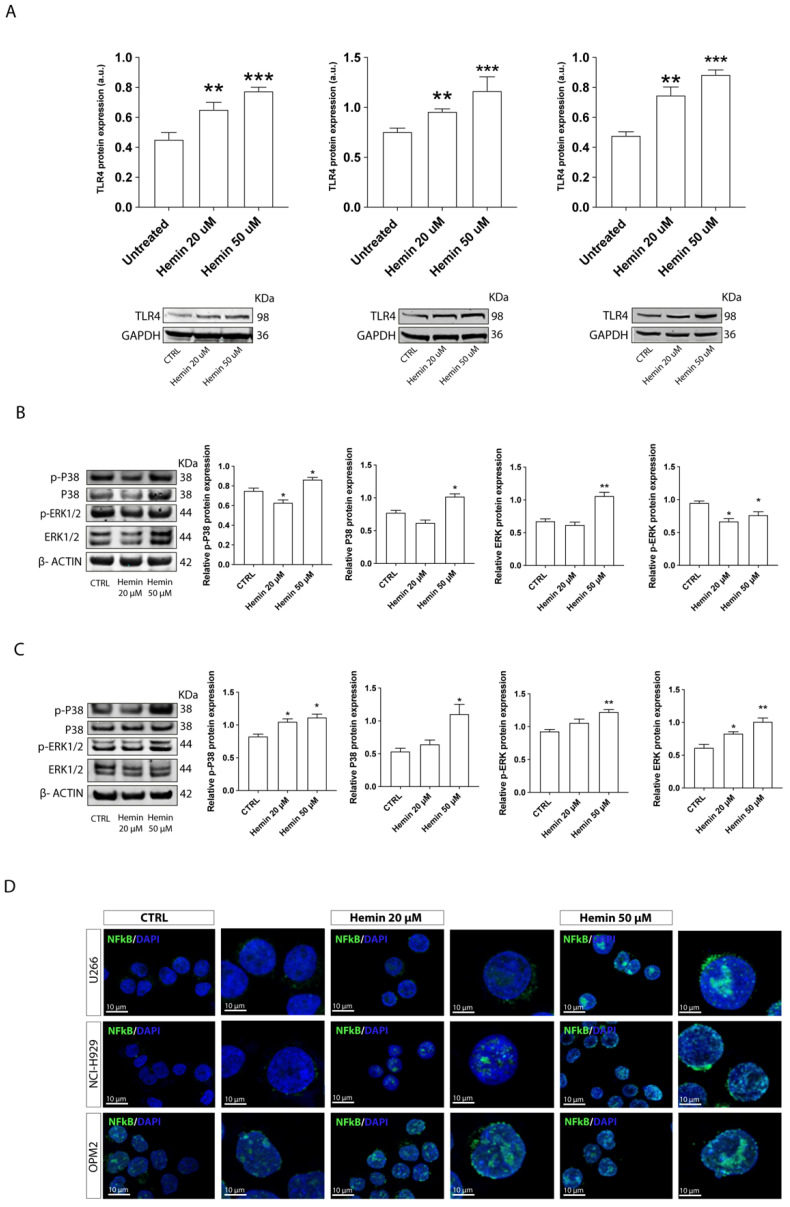
Hemin treatment increased TLR4 expression in myeloma cells. (**A**–**C**) Western blot analysis of TLR4 and MAP kinase P-38 and ERK1/2 expression after hemin treatment. GAPDH or β-actin proteins were used as total protein loading reference. For analysis, the optical density of the bands was measured using Scion Image software. (**D**) Immunofluorescence of Nrf2 (green) nuclear translocation after hemin treatment. Data are expressed as mean MFI ± SEM of *n* ≥ 4 biological replicates; * *p* < 0.05; ** *p* < 0.01; *** *p* < 0.001.

In a final series of experiments, we wanted to clarify whether HO-1 could regulate TLR4 expression in myeloma PCs. First, HMCLs were treated for 24 h with 20 µM or 50 µM hemin, an inducer of HO-1 protein expression and substrate of HO-1 enzymatic activity. Our results indicate that hemin significantly enhanced in a dose-dependent manner the levels of TLR4 protein expression (*p* < 0.01 and *p* < 0.001, respectively, with 20 µM and 50 µM for all HMCLs; Figure 7A). The expression of p38 and p-p38 also significantly augmented in MM cells after 50 µM hemin exposure (*p* < 0.05 compared to controls), while the increase in ERK and p-ERK was observed only in NCI-H929 cell line (*p* < 0.01) (Figure 7B,C). In U266 cells treated with 50 µM hemin, we found a lower p-ERK (*p* < 0.05) associated with an increase of ERK (*p* < 0.01) compared to untreated cells. To better investigate whether hemin activates MyD88-dependent TLR4 pathway, we next evaluated the activation of NF-kB. A significant increase in NF-kB nuclear translocation was observed both in U266 and NCI-H929 cells (Figure 7D). To further dissect the interplay standing between TRL4 and HO-1, we performed HO-1 silencing by short hairpin RNA. Our results showed that shHO-1 hampers TLR4 expression and its signaling pathway activation after LPS exposure (Figure S1B,C). As already described, TRL4 activation promotes MM cell proliferation. Therefore, to evaluate the functional inhibition of TLR4 after HO-1 silencing, we supplemented LPS for 24 and 48 h. Our results showed an impaired LPS-induced proliferation in shHO-1/U266 compared to the wild-type counterpart, overall corroborating our data proposing the outstanding role of HO-1/TRL4 axis in MM cells (Figure S1D).

**Figure 8:**
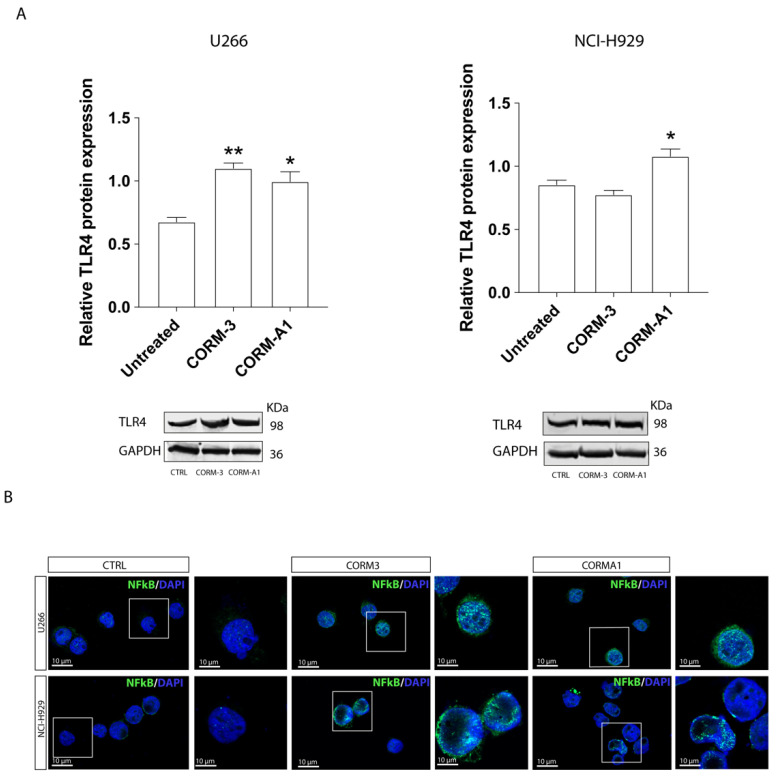
Carbon monoxide exposure induced TLR4 expression. (**A**) Western blot analysis of TLR4 expression after CORM-3 and CORM-A1 treatment in myeloma cells. GAPDH protein was used as total protein loading reference. For analysis, the optical density of the bands was measured using Scion Image software. (**B**) Immunofluorescence of Nrf2 (green) nuclear translocation after CORM-3 or CORM-A1 treatment. Data are expressed as mean MFI ± SEM of *n* ≥ 4 biological replicates; * *p* < 0.05; ** *p* < 0.01.

Finally, to better investigate the direct involvement of the HO-1/CO pathway in the modulation of TLR4 expression, we performed experiments in cells treated with the CO-releasing molecules CORM-A1 and CORM-3. We found that MM cells treated for 3 h with CORM-A1 (25 µM) or CORM-3 (10 µM) resulted in a significant increase in TLR4 protein levels (Figure 8A). We also found that the trafficking of NF-kB into the nucleus increased in MM cells after 24 h treatment with both CORM-3 and CORM-A1 (Figure 8B). Thus, these results indicate that CO produced by HO-1 may contribute to the resistance of MM cells to BTZ.

## 4. Discussion

The proteasome inhibitor BTZ is clinically used for the treatment of MM, but its efficacy is restricted by the widespread occurrence of resistance. Exposure to BTZ results in TLR4 activation (upregulation) in MM cells [4], leading to protection against BTZ-induced ER stress and ultimately favoring an anti-apoptotic effect through suppression of PERK/ATF4/CHOP branch [12], which are all part of a complex process known as UPR. ER stress influences many aspects of mitochondrial fitness and promotes mitochondrial depolarization and fragmentation [23]. Therefore, UPR activated during ER stress and mitochondrial plasticity become intrinsically associated with the response of myeloma PCs to BTZ. Notably, ER stress directly promotes HO-1 upregulation, which we reported increases in MM cells after BTZ treatment [16]. Such findings suggest that HO-1 maintains both mitochondrial fusion/fission and biogenesis/mitophagy homeostasis in astrocytes conferring protective effects against mitochondrial damage [24]. Recently, we demonstrated that TLR4 regulates mitochondrial biogenesis in myeloma PCs, and this pathway is upregulated in proteasome inhibitor-resistant cells [4]. Since TLR4 and HO-1/CO signaling pathways are both involved in the regulation of ER stress and mitochondrial dynamics, their interplay in response to BTZ treatment may reveal important molecular aspects to decipher the mechanisms of action underlying the resistance to this drug. To clarify how TLR4 and HO-1 pathways contribute together to BTZ resistance, we explored in MM cells the effects of BTZ in combination with either TAK-242 or SnPP, inhibitors of the TLR4 signaling and HO-1 enzymatic pathways, respectively. In accordance with our previous work, TAK-242/BTZ combination increased the efficacy of BTZ. This treatment increased the formation of ROS and induced higher mitochondrial depolarization leading to a significant reduction in mitochondrial mass, ultimately resulting in the upregulation of PINK1 and activation of mitophagy. The PINK1/Parkin axis is responsible for priming damaged mitochondria for selective autophagic recognition, a process known as mitophagy. In healthy mitochondria, PINK1 expression is low [22], whereas when mitochondria are damaged, PINK1 stabilizes on the outer membrane and recruits Parkin, thus allowing the efficient turnover of damaged mitochondria [25], which is dependent on the level of transmembrane potential across the inner membrane (Δψ_m_) [26]. Indeed, depolarization below a certain ∆Ψm is a prerequisite for the activation of a mitophagy that assures cell homeostasis; on the contrary, a strong mitochondrial damage leads to a collapse of mitochondrial dynamic balance with consequent cellular death [27]. Therefore, apoptosis observed in MM cells treated with TAK-242/BTZ in the present study is in part associated with a strong mitophagic response, which has been shown to facilitate cytochrome c release from mitochondria [28].

Our data also provide evidence that the combination of TAK-242 with BTZ decreased the activation of UPR survival pathway observed in MM cells treated with BTZ alone. GRP78, which is also referred to as BIP, is a major ER chaperone that controls the activation of UPR signaling [29]. Upon ER stress, GRP78 is released from ER transmembrane transducers, including PERK and IRE1α, leading to the activation of UPR survival pathways to block further damage. However, when the stress is too severe, the UPR triggers apoptotic responses [30]. TAK-242/BTZ treatment did not change the expression of GRP78 protein compared to BTZ alone, but significantly inhibited the upregulation both of PERK and IRE1α, leading to activation of CHOP, one of the UPR downstream effectors that promotes apoptosis. Concomitant with downregulation of PERK, we demonstrated a significant inhibition of BTZ-induced HO-1 upregulation in TAK-242/BTZ-treated cells. These results are consistent with the set of experiments demonstrating that TLR4 activation by LPS-induced Nrf2/HO-1 signaling in MM cells. PERK plays a key role in the adaptation of cells to ER stress and phosphorylates the α-subunit of eukaryotic translation initiation factor 2 (eIF2α) and Nrf2. Phosphorylated eIF2α attenuates protein translation relieving the burden on the ER, while phosphorylation of Nrf2 leads to its dissociation from Keap1, thus enabling Nrf2 to translocate to the nucleus where it acts on antioxidant response elements (AREs) that control the expression of several antioxidant genes [31]. The consequent HO-1 upregulation leads to activation of antioxidant and UPR survival pathway. In line with this scenario, we found that combination of BTZ with the inhibitor of HO-1 enzymatic activity SnPP decreased UPR and improved BTZ efficacy. Moreover, SnPP/BTZ treatment caused a higher mitochondrial depolarization associated with a reduction of mitochondrial mass compared to BTZ alone. Interestingly, NCI-H929 cells treated with SnPP/BTZ also showed lower BTZ-induced TLR4 upregulation, suggesting that products of HO-1 enzymatic activity could contribute to regulation of TLR4 expression by BTZ. This hypothesis is further supported by our data showing that MM cells treated with hemin or CO, a substrate and a by-product, respectively, of HO-1 enzymatic activity, increased TLR4 expression and activated its downstream signaling. Since stimulation of TLR4 induces Nrf2 nuclear translocation as well as its downstream antioxidant enzyme HO-1 in myeloma PCs, the current study supports the notion that TLR4 and HO-1 signaling pathways, both upregulated after BTZ treatment, activated each other. Consistently with our results, in macrophages LPS-mediated increase in HO-1 is dependent by increased levels of ROS promoting nuclear translocation of Nrf2 [32], which leads to HO-1 upregulation and its enzymatic by-products (i.e., CO, bilirubin/biliverdin) with consequent resolution of inflammation. In contrast to an inflammatory state, the end-product of HO-1 enzymatic activity CO contributes to decreased oxidative stress and ER stress, but also potentiates TLR4 pro-inflammatory signaling.

## 5. Conclusions

In conclusion, the present study demonstrates that treatment of MM cells with BTZ results in a cross-regulation between TLR4 and HO-1/CO signaling pathways, thus serving as a stress-responsive mechanism to increase UPR response and finally protecting mitochondria against the cytotoxic effects of BTZ and thus promoting drug resistance.

## Data Availability

The data presented in this study are available in the article and Supplementary Material.

## Supplementary Materials

The following supporting information can be downloaded at https://www.mdpi.com/article/10.3390/antiox11040767/s1, Figure S1: HO-1 knockdown impairs TLR4 expression and its signalling. (A,B) HO-1 and TLR4 expression analysis by qPCR analysis. shRNA against HO-1 efficiently mediate its knockdown. In parallel also TLR4 expression turns to be downregulated (C) NF-kB nuclear traslocation analysis by immunofluorescence. Upon HO-1 silencing, Nf-kB nuclear translocation turns to be imapired. (D) XTT assay on shHO-1 cells. Cells transfected with shRNA against HO-1 did not respond to LPS-induced proliferation as control cells [33,34].
