# Supplementary material for: TLR4 Signaling and Heme Oxygenase-1/Carbon Monoxide Pathway Crosstalk Induces Resiliency of Myeloma Plasma Cells to Bortezomib Treatment
Source: Antioxidants (Basel). 2022 Apr 12;11(4):767. doi: 10.3390/antiox11040767 (PMC9031632; doi:10.3390/antiox11040767)
Supplement: Supplementary file 1 [file antioxidants-11-00767-s001.zip › antioxidants-1652539-supplementary.pdf]

antioxidants

Article

TLR4 Signaling and Heme Oxygenase-1/Carbon Monoxide Pathway Crosstalk Induces Resiliency of Myeloma Plasma Cells to Bortezomib Treatment

Grazia Scandura [1,†], Cesarina Giallongo [2,†], Fabrizio Puglisi [1], Alessandra Romano [1], Nunziatina Laura Parrinello [1], Tatiana Zuppelli [3], Lucia Longhitano [3], Sebastiano Giallongo [3], Michelino Di Rosa [3], Giuseppe Musumeci [3], Roberto Motterlini [4], Roberta Foresti [4], Giuseppe Alberto Palumbo [2,*], Giovanni Li Volti [3,*], Francesco Di Raimondo [1,‡] and Daniele Tibullo [3,‡]

1 Division of Hematology, Department of General Surgery and Medical-Surgical Specialties, A.O.U. "Policlinico-Vittorio Emanuele", University of Catania, 95123 Catania, Italy; gra.scandura@gmail.com (G.S.); puglisi.fabri@gmail.com (F.P.); sandrina.romano@gmail.com (A.R.); lauraparrinello@tiscali.it (N.L.P.); diraimon@unict.it (F.D.R.)

2 Department of Scienze Mediche Chirurgiche e Tecnologie Avanzate "G.F. Ingrassia", University of Catania, 95123 Catania, Italy; cesarina.giallongo@unict.it

3 Department of Biomedical and Biotechnological Sciences, University of Catania, 95123 Catania, Italy; tatiana.zuppelli@gmail.com (T.Z.); lucialonghitano@hotmail.it (L.L.); sebastiano.giall@gmail.com (S.G.); mdirosa@unict.it (M.D.R.), g.musumeci@unict.it (G.M.); d.tibullo@unict.it (D.T.)

4 Faculty of Health, University Paris-Est Creteil, INSERM, IMRB, 94010 Creteil, France; roberto.motterlini@inserm.fr (R.M.); roberta.foresti@inserm.fr (R.F.)

* Correspondence: palumbo.gam@gmail.com (G.A.P.); livolti@unict.it (G.L.V.)

† These authors equally contributed to this work.

‡ These authors equally contributed to this work.

Citation: Scandura, G.; Giallongo, C.; Puglisi, F.; Romano, A.; Parrinello, N.L.; Zuppelli, T.; Longhitano, L.; Giallongo, S.; Di Rosa, M.; Musumeci, G.; et al. TLR4 Signaling and Heme Oxygenase-1/Carbon Monoxide Pathway Crosstalk Induces Resiliency of Myeloma Plasma Cells to Bortezomib Treatment. *Antioxidants* **2022**, *11*, 767. https://doi.org/10.3390/antiox11040767

Academic Editor: Stanley Omaye

Received: 9 March 2022
Accepted: 8 April 2022
Published: 12 April 2022

Publisher's Note: MDPI stays neutral with regard to jurisdictional claims in published maps and institutional affiliations.

Copyright: © 2022 by the authors. Licensee MDPI, Basel, Switzerland. This article is an open access article distributed under the terms and conditions of the Creative Commons Attribution (CC BY) license (https://creativecommons.org/licenses/by/4.0/).

Supplemental Material.

1. Supplemental Method

shHO-1 MM cells generation and XTT proliferation assay

U266 cells were silenced for HO-1 as previously described [1]. To assay their proliferation cells were plated in a 96-well plate and treated with 2 ug/mL LPS for 24 and 48 hours. XTT assay was then performed as already reported[2].

Supplemental Figures

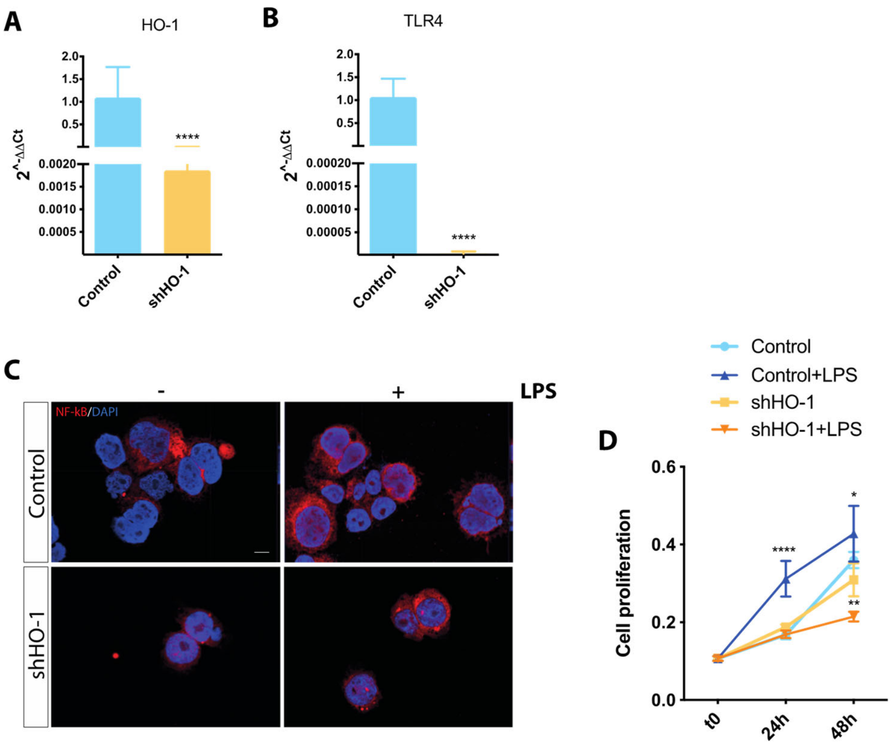

Figure S1. HO-1 knockdown impairs TLR4 expression and its signalling. (**A-B)** HO-1 and TLR4 expression analysis by qPCR analysis. shRNA against HO-1 efficiently mediate its knockdown. In parallel also TLR4 expression turns to be downregulated (**C)** NF-kB nuclear traslocation analysis by immunofluorescence. Upon HO-1 silencing, Nf-kB nuclear translocation turns to be imapired. (**D)** XTT assay on shHO-1 cells. Cells transfected with shRNA against HO-1 did not respond to LPS-induced proliferation as control cells.

Supplemental References

1. Tibullo, D.; Barbagallo, I.; Giallongo, C.; La Cava, P.; Parrinello, N.; Vanella, L.; Stagno, F.; Palumbo, G.A.; Li Volti, G.; Di Raimondo, F. Nuclear translocation of heme oxygenase-1 confers resistance to imatinib in chronic myeloid leukemia cells. *Curr Pharm Des* **2013**, *19*, 2765-2770, doi:10.2174/1381612811319150012.
2. Maugeri, G.; D'Amico, A.G.; Giunta, S.; Giallongo, C.; Tibullo, D.; Bucolo, C.; Saccone, S.; Federico, C.; Scollo, D.; Longo, A.; et al. Activity-Dependent Neuroprotective Protein (ADNP)-Derived Peptide (NAP) Counteracts UV-B Radiation-Induced ROS Formation in Corneal Epithelium. *Antioxidants (Basel)* **2022**, *11*, doi:10.3390/antiox11010128.
